# Sustainable development of smart cities and smart territories based on the model of minimizing externalities

**DOI:** 10.12688/f1000research.114630.1

**Published:** 2022-05-13

**Authors:** Guldana Kuandykovna Suyendikova, Sergey Evgenievich Barykin, Sergey Mikhailovich Sergeev, Irina Vasilievna Kapustina, Yuri Krupnov, Natalia NikolaevnaShchepkina

**Affiliations:** 1 Department of Economics, L.N. Gumilyov Eurasian National University, Nur-Sultan, 010008, Kazakhstan; 2 Graduate School of Service and Trade, Peter the Great St. Petersburg Polytechnic University, St. Petersburg, 195251, Russian Federation; 3 Graduate School of Industrial Management, Peter the Great St. Petersburg Polytechnic University, St. Petersburg, 195251, Russian Federation; 4 Center for Strategic Forecasting and Planning, Financial University under the Government of the Russian Federation, Moscow, 124167, Russian Federation; 5 Department of construction and real estate management, Moscow State University of Civil Engineering, Mowcow, 109377, Russian Federation

**Keywords:** Smart Cities, Digital Platforms, Sustainable Development, Digital Interactions, Smart Territories, Agglomeration, Satellite-Cities.

## Abstract

The development of conceptual models of a digital city poses numerous challenges for developers. The public sector concept model has become one of the most difficult models to use. When developing algorithms to find a solution, the multidirectional interests of businesses and public institutions are combined. This type of model reflects the most acute and urgent problems faced by megapolises with regard to combining numerous localized services provided to the community in a limited territory. The administrations of both cities and regions (the scale of the smart territories) must make decisions concerning overcoming the barriers existing between the profits of commercial structures, the negative externalities generated by their activities, and the social benefits to the population in the territory under their control. It is necessary to solve this problem to achieve the effective management of enterprises belonging to the segment of long-term participants in various business activities, interacting with the surrounding social and business environment in a complex. This study takes into account the complex structures of the economic processes characteristic of megacities. The periodicity of economic processes is also taken into account. When choosing an optimization criterion, functions reflecting the level of internalization of responsibility for external effects were considered. The authors propose a mathematical model that can be used as part of the management decision support systems software, aiming at taking into account the externalities of a wide range of national, institutional, business, and social activities.

## Introduction

Several possible actions from a comprehensive perspective could be significant in Post-COVID recovery. ^1^^–^^4^ The state as a whole, from the point of view of the economy, is a set of enterprises, both institutional and commercial. When studying economic processes at the top level, profits, common interests, and obligations must be taken into account. ^5^^,^^6^ Additionally, it is important to introduce formalisms for the effect of externalities, which occur everywhere. Besides entrepreneurial activity, state institutions of the defense department, medicine, transport, education, etc., operate in each country. When modeling externalities, it is important to take into account both their positive and negative aspects based on the activities of all market entities.

Any reaction can be the genesis in the general case, and therefore it is necessary to not be limited to the field of ecology in smart cities and in the smart territories considering the larger scale. ^7^ The consequences of a social nature are much wider and deeper. ^8^ Such damage to society will first be taken into account when monitoring the economic indicators of the current activities of any market participants.

As a rule, such analysis uses the methods of surrogate markets. In turn, this direction, which is usually referred to as an indirect market method or proxy market, is divided into several pricing principles.

Since, at present, there is no definitive methodology able to theoretically cover the problem in general, we will consider the dependencies of externalities in the form of a function, the arguments concerning which are the characteristics of economic activity. ^9^ An additional circumstance complicating any activity is the uncertainty of market conditions. Here, the methods of the stochastic process theory are used, which makes it possible to form adequate mathematical models. ^10^ However, it must be taken into account that the indicators of distribution functions are not static. In the process of solving the problem, a set of algorithms are defined and the economic criteria used is justified. When evaluating a set of planned calculation results, special attention should be paid to finding a balance between the additional burden on the enterprise—i.e., on internalization and the indicators of the profitability of its activities. Based on this set of conditions, the formalisms of the mathematical model should be built. Similarly, non-commercial activities must be accounted for. This is necessary in order to assess the social factor in the activities of state institutions themselves in terms of their social and economic efficiency.

In this case, optimization is carried out by searching for the extrema of functionality, taking into account the inclusion of externalities in the market mechanism for evaluating performance. The results obtained may be a set of regulatory measures using institutional mechanisms. These include dispositive, strategic, restrictive, and stimulating components that form the basis of administrative management and legislative acts that are implemented in both the concepts of preventive behavior and prevention of damage, as well as in the more general concepts of the preservation and development of the social environment. ^11^

## Methods

Improving the smart city concept is essential to meet the demand of growing urban conglomerates to maintain comfort ^12^ and improve the quality of urbanization. ^13^^,^^14^ Regulation of the internal flows of megacities is the main focus of maintaining the quality of urbanization. ^15^ Ignoring environmental requirements to reduce landscape characteristics, which will occur without striving to maintain the structure and functions of the regional ecological system. ^16^ Urbanization policy is closely related to a wide range of objectives, including transport policy, the provision of public infrastructure, and the provision of modern security and management facilities. ^17^ Urbanization affects the condition and viability of green infrastructure and its maintenance as a source of ecosystem services, which will allow the development of effective policies for land use, sustainable urban development and infrastructure management. ^18^ A recent study suggested the sustainable development of smart cities as a complex structure of interconnected organizations that influence the level of everyday life of the population. ^19^

The authors developed a formalized description to solve the problem at hand. The methods presented in the literature operate, as a rule, with the tools of correlation and regression analysis. ^20^ To find the optimal solution under the conditions of market uncertainty and to apply the optimization methods correctly, a more complex mathematical model is needed.

Since the processes of economic activity have some duration in terms of time and also have a complex nature based on changes in seasonal indicators, the authors used a combination of methods. ^21^ Among them, we note the theory of the calculus of variations, methods for solving differential equations, the theory of mathematical games, and the main provisions ^5^^,^^6^ of methods used for finding optimal control. ^22^^,^^23^

## Results

In order to describe the processes under study, we introduce a number of parameters that describe business and government activities. The mathematical model we propose is based on the application of methods for finding optimal solutions.

We introduce the concept of the number ( N ) of enterprises. All of them work in this limited area. Let us take into account the fact that these enterprises have negative externalities as an external influence. In the case of additional costs for each enterprise, the effect of negative externalities can be reduced.

We denote $\bar{P}=\left(p_{1},p_{2},\ldots ,p_{N}\right)$ as the vector of searching for the optimal equilibrium solution. Searching for options for such solutions is carried out inside an N -dimensional cube of economic situations. We make the calculation specific for N=3 , since in this case the result can be visualized. Let us enter the value of additional expenses ω . This amount reflects the need to spend additional money when planning work aimed at minimizing damage from the externalities produced.

Next, we take into account the possible differences in the scale of enterprises, such as the differences in damage $\psi^{*}$ and ψ . We summarize the calculation model and data in Table 1.

**Table 1.** Variants of situations and the calculation model.

Option	Enterprise			Calculation
	I	II	III	Element value
	Expenses			
0,0,0	ω	ω	ω	$\left(1-p_{1}\right)\left(1-p_{2}\right)\left(1-p_{3}\right)$
0,0,1	ω	ω	0	$\left(1-p_{1}\right)\left(1-p_{2}\right)p_{3}$
0,1,0	ω	0	ω	$\left(1-p_{1}\right)p_{2}\left(1-p_{3}\right)$
0,1,1	$\psi^{*}$ + ω	$\psi^{*}$	$\psi^{*}$	$\left(1-p_{1}\right)p_{2}p_{3}$
1,0,0	0	ω	ω	$p_{1}\left(1-p_{2}\right)\left(1-p_{3}\right)$
1,0,1	ψ	ψ + ω	ψ	$p_{1}\left(1-p_{2}\right)p_{3}$
1,1,0	ψ	ψ	ψ + ω	$p_{1}p_{2}\left(1-p_{3}\right)$
1,1,1	$\psi^{*}$	$\psi^{*}$	$\psi^{*}$	$p_{1}p_{2}p_{3}$

To calculate, we assume that ${\bar{p}}_{i}=1-p_{i}$ , ∀i and calculate the product of the vector below:

$$\left({\bar{p}}_{1}{\bar{p}}_{2}{\bar{p}}_{3},{\bar{p}}_{1}{\bar{p}}_{2}p_{3},{\bar{p}}_{1}p_{2}{\bar{p}}_{3},{\bar{p}}_{1}p_{2}p_{3},p_{1}{\bar{p}}_{2}{\bar{p}}_{3},p_{1}{\bar{p}}_{2}p_{3},p_{1}p_{2}{\bar{p}}_{3},p_{1}p_{2}p_{3}\right)$$ (1)

using the corresponding vectors in Table 1. We write these out in the following form:

$$\begin{array}{c} \left(\omega ,\omega ,\omega ,\psi^{*}+\omega ,0,\psi ,\psi ,\psi^{*}\right);\left(\omega ,\omega ,0,\psi^{*},\omega ,\psi +\omega ,\psi ,\psi^{*}\right);\left(\omega ,0,\omega ,\psi^{*},\omega ,\psi ,\psi +\omega ,\psi^{*}\right). \end{array}$$

By applying the solution-finding rule ^24^ to the formulated conditions, we obtain two inequalities. The calculated ratios reflect possible market equilibrium conditions. First, we need to define the conditions for the lower bound:

$$-\omega \left(1-p_{1}\right)\left(1-p_{2}\right)\left(1-p_{3}\right)-\omega \left(1-p_{1}\right)\left(1-p_{2}\right)p_{3}-\omega \left(1-p_{1}\right)p_{2}\left(1-p_{3}\right)-\left(\psi^{*}+\omega \right)\left(1-p_{1}\right)p_{2}p_{3}-\psi p_{1}\left(1-p_{2}\right)p_{3}-\psi p_{1}p_{2}\left(1-p_{3}\right)-\psi^{*}p_{1}p_{2}p_{3}\geq -\omega \left(1-p_{2}\right)\left(1-p_{3}\right)-\omega p_{2}\left(1-p_{3}\right)-\omega \left(1-p_{2}\right)p_{3}-\left(\psi^{*}+\omega \right)p_{2}p_{3}.$$

The next calculation step allows us to determine the upper limit:

$$-\omega \left(1-p_{1}\right)\left(1-p_{2}\right)\left(1-p_{3}\right)-\omega \left(1-p_{1}\right)\left(1-p_{2}\right)p_{3}-\omega \left(1-p_{1}\right)p_{2}\left(1-p_{3}\right)-\left(\psi^{*}+\omega \right)\left(1-p_{1}\right)p_{2}p_{3}-\psi p_{1}\left(1-p_{2}\right)p_{3}-\psi p_{1}p_{2}\left(1-p_{3}\right)-\psi^{*}p_{1}p_{2}p_{3}\geq -\psi p_{2}\left(1-p_{3}\right)-\psi \left(1-p_{2}\right)p_{3}-\psi^{*}p_{2}p_{3}.$$

We then carry out simple transformations and obtain the system:

$$\left.\begin{array}{c} \omega p_{1}-\psi p_{1}p_{2}-\psi p_{1}p_{3}+2\psi p_{1}p_{2}p_{3}\geq 0\\ -\omega \left(1-p_{1}\right)+\psi \left(p_{2}+p_{3}\right)\left(1-p_{1}\right)-2\psi p_{2}p_{3}\left(1-p_{1}\right)\geq 0 \end{array}\right\}$$ (2)

The calculation process used for each enterprise is similar. As a result, one obtains a system of equations for calculating the boundaries necessary for making decisions:

$$\left.\begin{array}{c} \omega p_{2}-\psi^{*}p_{2}p_{3}-\psi p_{1}p_{2}+2\psi p_{1}p_{2}p_{3}\geq 0\\ \omega \left(1-p_{2}\right)-\psi^{*}\left(1-p_{2}\right)p_{3}-\psi p_{1}\left(1-p_{2}\right)+2\psi p_{1}\left(1-p_{2}\right)p_{3}\geq 0\\ \omega p_{3}-\psi^{*}p_{2}p_{3}-\psi p_{1}p_{3}+2\psi p_{1}p_{2}p_{3}\geq 0\\ \omega \left(1-p_{3}\right)-\psi^{*}\left(1-p_{3}\right)p_{2}-\psi p_{1}\left(1-p_{3}\right)+2\psi p_{1}\left(1-p_{3}\right)p_{2}\geq 0 \end{array}\right\}$$ (3)

**Figure 1.**
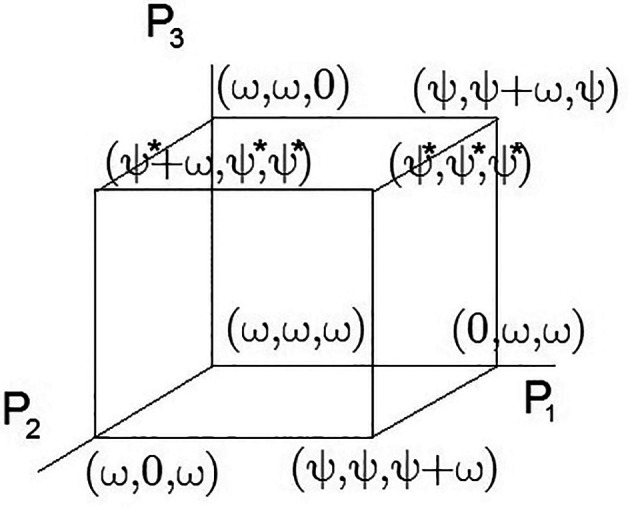
A 3D set of admissible solutions.

All possible solutions are limited within the multidimensional cube of situations. Such visualization is applicable to the three-dimensional case considered in this example and is used solely for clarity. In the case of N=3 , this can be visualized in Figure 1. At the cube corners, the economic indicators associated with externalities are marked.

As a result of solving the system of equations, we obtain a set of regions for $\left\{p_{1},p_{2},p_{3}\right\}$ . The vector values belong to the multidimensional space of situations that satisfy the Nash equilibrium condition. The obtained data is represented in the simplest way by constructing volumetric diagrams of the solution in any package of mathematical applied programs. An analysis of the obtained equations shows that the domains of admissible solutions belong to the intersection of planes with hyperbolic surfaces.

**Figure 2.**
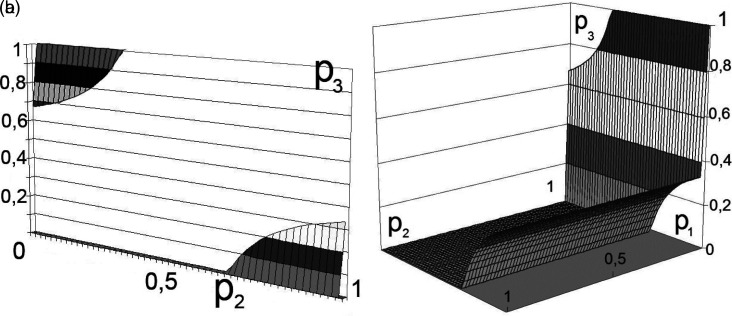
a) Boundary solution areas. b) Set of solutions for Equation (2).

**Figure 3.**
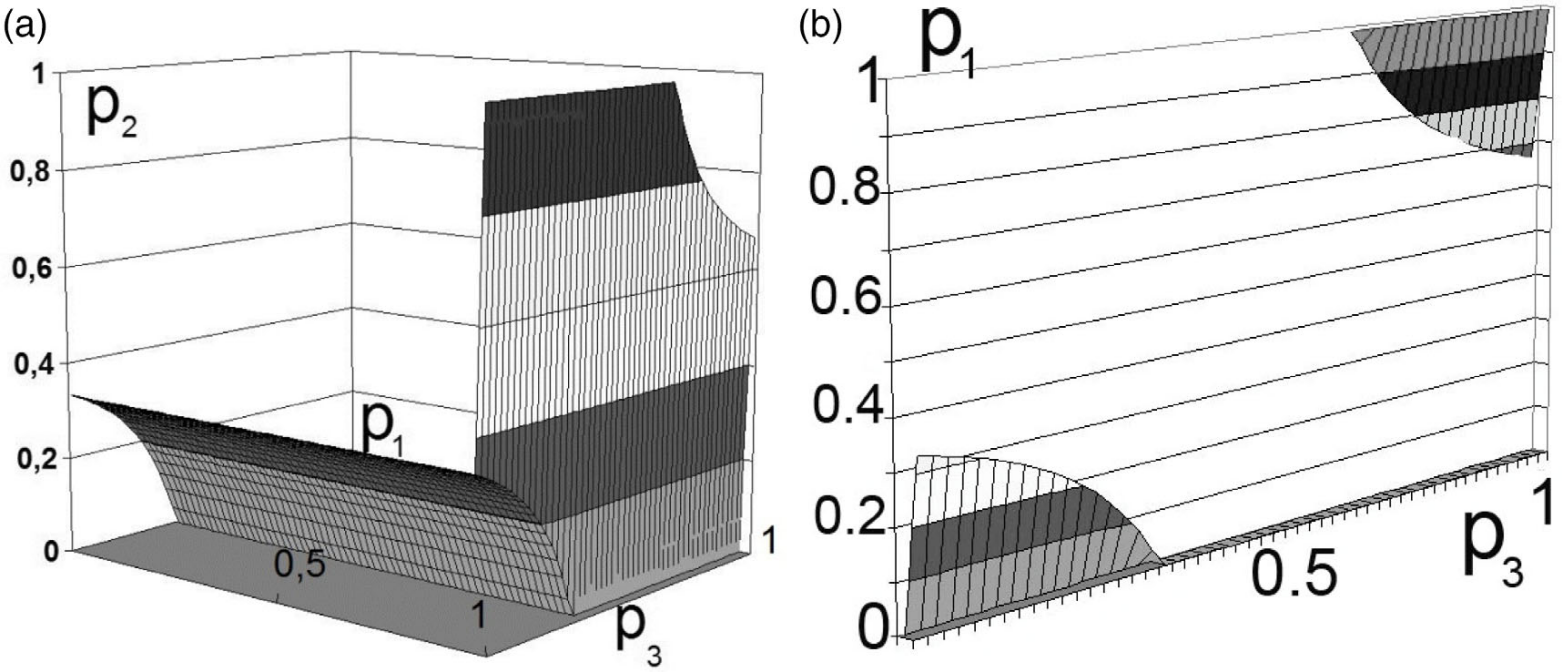
a) Set of solutions for Equation (3). b) Boundary solution areas.

The calculation results for the two participants in production activities are shown in Figures 2 and 3. ^38^ The third solution differs only in terms of the orthogonal rotation of the axes in which the diagram is built.

Note that the equations are hyperbolic surfaces, with several intersection points giving the desired solution. To do this, it is sufficient, for example, to transform Equations (2) and (3) to the following form:

$$P_{2}\geq \frac{1}{2}-\frac{\psi \left(2\omega /\psi -1\right)}{\psi P_{3}-\psi /2}.$$

In this case, the variation in the boundaries forms multidimensional dependencies, as presented in Figures 2 and 3.

The first version of the result reflects a trivial solution $\left({\bar{p}}_{1}{\bar{p}}_{2}{\bar{p}}_{3}\right)=0$ . In addition to this case, it is possible to obtain stable states in two more variants. The calculation of the second vector makes it possible to determine the components that satisfy the equilibrium conditions:

$$\left\{\frac{\omega -\psi \frac{\psi +\sqrt{\psi^{2}-2\omega \psi }}{2\psi }+2\psi {\left[\frac{\psi +\sqrt{\psi^{2}-2\omega \psi }}{2\psi }\right]}^{2}}{\psi {\left[\left(\psi +\sqrt{\psi^{2}-2\omega \psi }\right)/2\psi \right]}^{2}},\frac{\psi +\sqrt{\psi^{2}-2\omega \psi }}{2\psi },\frac{\psi +\sqrt{\psi^{2}-2\omega \psi }}{2\psi }\right\}$$

Let us calculate the third case of equilibrium in a similar way. The desired vector is equal to:

$$\left\{\frac{\omega -\psi \frac{\psi -\sqrt{\psi^{2}-2\omega \psi }}{2\psi }+2\psi {\left[\frac{\psi -\sqrt{\psi^{2}-2\omega \psi }}{2\psi }\right]}^{2}}{\psi {\left[\left(\psi -\sqrt{\psi^{2}-2\omega \psi }\right)/2\psi \right]}^{2}},\frac{\psi -\sqrt{\psi^{2}-2\omega \psi }}{2\psi },\frac{\psi -\sqrt{\psi^{2}-2\omega \psi }}{2\psi }\right\}$$

## Discussion

### Analysis of the results

Each calculated value is applied in different conditions. For the administration of cities and regions, the decision is made in order to overcome a number of barriers. This applies primarily to the disagreement between the profits of commercial structures, the negative externalities generated by their activities, and the social benefit of the population in the controlled territory. In the case of the dispositive method of legal regulation, the first (trivial) solution is applied everywhere.

This decision (presented in Figures 2 and 3) can be interpreted as the unwillingness of the participants in production activities to bear the costs of transforming external effects into internal ones. ^25^

The second equilibrium solution accounts for restrictive measures. This approach requires the application of regulatory standards.

The third result of the decision involves the application of radical measures of restriction.

The listed measures have an economic character. The application of these restrictions obliges economic entities to conduct their activities while taking into account the interests of society. This will also generally affect the state of the entire economic system. ^26^^–^^30^

It should be emphasized that the presented equations of the mathematical model reflect the situation for N=3 participants in commercial activities. This is explained by the fact that, in this case, it is possible to visualize the calculation results. The equations developed for the mathematical model can be scaled. At the same time, the number of participants ^31^ in economic activities is not limited. In addition, the dependencies describing economic indicators can also have an arbitrary form. This only increases the dimension of the externalities model. All calculations were implemented using Microsoft Excel (Microsoft, 2022) (RRID:SCR_016137).

### Application of results

The application of the obtained results in the organization of the life of modern megapolises is especially relevant. Due to the aggravated environmental situation, problems of both a social and economic nature are actively manifested in them. The effective work of the authorities will be based on a scientifically grounded methodology for solving problems related to the economics of the environment. Therefore, the efficient use of public resources is emphasized as among the major tasks that must be completed. The task of business analysis aimed at developing recommendations for authorities and governments is to take into account multidirectional processes. On the one hand, there is an increased burden on resources and a decrease in the quality indicators of these resources, and it is necessary to evaluate the negative externalities. For the economic indicators of the metropolis, the standard ^26^ of living of the population depends on the activities of all types of businesses and on enterprises that create profit and employment. The results presented by the authors of this paper and the mathematical model ^32^^,^^33^ make it possible to develop a solution algorithm. Based on this, it is possible to create expert systems. The development of large metropolitan areas and industrial centers is accompanied by data exchange flows. Modern big data technologies and statistical analysis provide operational economic information for calculations based on mathematical models. Such systems are promising for use in the environmental, social, and corporate management of a smart city at the top level of planning.

### Structure of relationships

High rates of urbanization have caused large-scale shifts in the entire structure of relationships (relationships between business entities operating in a given territory, administration, and the population as a user of public resources). The dynamics of the mutual influence of different types of activity have intensified. Over the past decade, the world has come to increasingly rely on scientific and technological achievements. This inevitably entails negative consequences, which, in conditions with a high concentration of population and industry, inevitably create problems of both a social and economic orientation. The nature of these externalities is not determined solely by their impact on the environment. The quality of life in general is also negatively affected. The desire of the population to move to megapolises is determined by the high-quality standards of the living environment, and if radical measures are not taken to regulate the entire infrastructure, we will see the opposite effect. The functioning of numerous social institutions, public utilities, the service sector, and the industrial sector should be coordinated within smart city digital platforms.

The development of algorithms for making intelligent decisions is only possible today by combining digital data flows, big data technologies, and information communications into a single system. Decision criteria can be multifaceted. Science-based accounting of the balance between profit affecting the welfare and minimizing the negative impact of industrial urbanization is needed. The solution to socio-territorial problems depends on the quality of management decision-making algorithms. These should be based on mathematical models that are close to reality and methods for finding optimal solutions.

## Conclusion

Urbanization reflects a global trend. Consolidation into large megapolises is based on a multifaceted process involving the development of society as a whole. Megapolises, alpha cities, and the neighborhoods of such agglomerations, at present, house up to half of the world’s population and the majority of industrial enterprises. The smart city concept has no alternative today. The set of expert algorithms within the framework of the smart city conceptual model is intended primarily for decision-makers in each of the sectors of the economy. As a result, the development of directives for business organizers, systems, and services necessary for a megapolis is carried out based on calculated and economically sound principles. In many ways, the work of the e-government is guided by similar principles. The main principles are still the commitment to sustainable development ^34^ and maintaining the quality of life of the population. ^27^^,^^35^ The authors propose a complex approach to consider the socially oriented combination of ICT (information, and communication technology) tools for the rational use of resources to improve life quality indicators. The authors are attempting to develop smart city concept considering the public sector concept model (PSCM). The authors’ recommendations aimed at organizations that provide services and manage data in cities. The proposed approach addresses the interoperability of systems and data-sharing so that information from different sources can be normalized, classified, shared and understood, with the derivation of data linked back to previous layers and the impact of decisions observable in operational data. The stated principles of formalization are the basis for the development of a mathematical model. The use of the decision algorithm serves as a rationale for making a number of management decisions. At present, the concentration of business and cultural activity on a limited territorial scale dominates. This gives rise to the need to determine the feasibility of internalizing the numerous effects that arise from business or governmental activities. The presented technique makes it possible to formalize these according to the principle of externalities and to apply a multidimensional balance calculation to minimize the possible damage caused. It is necessary for management structures ^36^ or administration bodies to exclude decision-making ^37^ based on heuristic methods. It is necessary to carry out the analysis on a verified, scientifically based calculation. The result of mathematical modeling will be the optimization of the amount of expenses that must be borne by various members of the business community. It should be noted that today, in the decision-making process, dynamic analyses of the situation in the economy using digital twins are not carried out and methods for finding optimal solutions are not applied. All of these shortcomings occur for many reasons. These include the imperfection of methods and the complexity of taking into account many factors. We would also point to the lack of correct theoretical models and digital twins of processes in megacities based on accounting for economic indicators.

## Data availability

### Underlying data

Figshare: Figures.xls https://doi.org/10.6084/m9.figshare.19692205.v1 ^38^

This project contains the following underlying data:

- Figures.xls (This is the data used for the calculations shown in this research paper). The EXCEL application contains the visualization of calculations according to the formulas presented in the work.

Data are available under the terms of the Creative Commons Attribution 4.0 International license (CC-BY 4.0).

## Ethical approval

Not applicable (No use of individual human data in this article).
