# Supplementary material for: Sustainable development of smart cities and smart territories based on the model of minimizing externalities
Source: F1000Res. 2022 Jun 6;11:522. Originally published 2022 May 13. [Version 2] doi: 10.12688/f1000research.114630.2 (PMC9171296; doi:10.12688/f1000research.114630.2)
Supplement: Peer review history

# Peer review: Reviewer response for version 2

**DOI:** 10.5256/f1000research.134552.r139693

**Published:** 2022-06-07

**Authors:** Mui Yee Cheok

**Affiliations:** 1 Universiti Tun Abdul Razak Kuala Lumpur, Kuala Lumpur, Malaysia

Suggestions have been done.

Is the work clearly and accurately presented and does it cite the current literature?

Yes

If applicable, is the statistical analysis and its interpretation appropriate?

Partly

Are all the source data underlying the results available to ensure full reproducibility?

Yes

Is the study design appropriate and is the work technically sound?

Yes

Are the conclusions drawn adequately supported by the results?

Yes

Are sufficient details of methods and analysis provided to allow replication by others?

Yes

Reviewer Expertise:

NA

I confirm that I have read this submission and believe that I have an appropriate level of expertise to confirm that it is of an acceptable scientific standard.

# Peer review: Reviewer response for version 2

**DOI:** 10.5256/f1000research.134552.r139690

**Published:** 2022-06-07

**Authors:** Jakub Kubiczek

**Affiliations:** 1 Department of Economic and Financial Analysis, University of Economics in Katowice, Katowice, Poland

Dear Authors,

 Thank you very much for the responses to my remarks, which were my only ones. The revised version is definitely better.

 Best regards,

 Jakub Kubiczek

Is the work clearly and accurately presented and does it cite the current literature?

Yes

If applicable, is the statistical analysis and its interpretation appropriate?

Partly

Are all the source data underlying the results available to ensure full reproducibility?

Partly

Is the study design appropriate and is the work technically sound?

Partly

Are the conclusions drawn adequately supported by the results?

Yes

Are sufficient details of methods and analysis provided to allow replication by others?

Partly

Reviewer Expertise:

Decision making, Corporate Social Responsibility, Sustainable Development

I confirm that I have read this submission and believe that I have an appropriate level of expertise to confirm that it is of an acceptable scientific standard.

# Peer review: Reviewer response for version 2

**DOI:** 10.5256/f1000research.134552.r139692

**Published:** 2022-06-06

**Authors:** Bilal Khalid

**Affiliations:** 1 KMITL Business School, King Mongkut's Institute of Technology Ladkrabang, Bangkok, Thailand

I am very satisfied with the improvements and present paper quality. Strongly support the paper's indexing.

Is the work clearly and accurately presented and does it cite the current literature?

Partly

If applicable, is the statistical analysis and its interpretation appropriate?

Yes

Are all the source data underlying the results available to ensure full reproducibility?

No

Is the study design appropriate and is the work technically sound?

Partly

Are the conclusions drawn adequately supported by the results?

Yes

Are sufficient details of methods and analysis provided to allow replication by others?

Partly

Reviewer Expertise:

Smart Cities, Sustainable Practices, De-carbonization, Immigrant Entrepreneurship, Global Value Chains, Industry 4.0, Digital Transformations, Technology Adoption, Consumer behavior, Public Policy,

I confirm that I have read this submission and believe that I have an appropriate level of expertise to confirm that it is of an acceptable scientific standard.

# Peer review: Reviewer response for version 1

**DOI:** 10.5256/f1000research.126380.r137830

**Published:** 2022-05-24

**Authors:** Esra Sipahi Dongul

**Affiliations:** 1 Faculty of Health Sciences, Department of Social Work, Aksaray University, Aksaray, Turkey

The authors talk about smart cities. Please elaborate with some concrete example not more than a paragraph.

- Please provide more detail about all of the methods used.
- Please specify what you mean when you use the term of the multidimensional cube of situations.
- Are the economic indicators associated marked with externalities?
- What is the sense of the expression 3? This need to be elaborated well.
- Please explain with concrete examples the theoretical base of the the mathematical model.
- Light up the term “green infrastructure”.

Is the work clearly and accurately presented and does it cite the current literature?

Yes

If applicable, is the statistical analysis and its interpretation appropriate?

Yes

Are all the source data underlying the results available to ensure full reproducibility?

Yes

Is the study design appropriate and is the work technically sound?

Yes

Are the conclusions drawn adequately supported by the results?

Yes

Are sufficient details of methods and analysis provided to allow replication by others?

Yes

Reviewer Expertise:

Business, management, organizational behavior

I confirm that I have read this submission and believe that I have an appropriate level of expertise to confirm that it is of an acceptable scientific standard, however I have significant reservations, as outlined above.

# Response

**Published:** 2022-05-28

**Authors:** Guldana Kuandykovna Suyendikova

**Affiliations:** Department of Economics, L.N. Gumilyov Eurasian National University, Russian Federation

Dear Esra Sipahi Dongul

 Thank you for your precious comments on our manuscript ‘ *Sustainable development of smart cities and smart territories based on the model of minimizing externalities’* Your recommendations helped polished the manuscript, and we also appreciate your full consideration to our paper.

 As per your instructions, we have included all of your comments points by point individually, indicating exactly how we addressed each concern or problem and describing the changes we have made. The revised manuscript was formatted according to the journal formatting criteria. We hope the revised manuscript will better suit F1000Research but are happy to consider further revisions if any, and we thank you for your interest in our research.

**REVIEWER COMMENTS, AUTHOR RESPONSES AND MANUSCRIPT CHANGES**

**Comment 1: **Please provide more detail about all of the methods used.

**Response: **Thank You! We have clarified the methods in the revised version.

**Comment 2: **Please specify what you mean when you use the term of multidimensional cube of situations.

**Response: **Thank you for your comment. We described the meaning of the multidimensional cube of situations.

**Comment 3**: Are the economic indicators associated marked with externalities?

**Response:** Thank You for highlighting this confusion. This confusion was removed and we described the logical connection between the economic indicators and externalities.

**Comment 4: **What is the sense of the expression 3? This need to be elaborated well.

**Response: **We elaborated the revised version in order to clarify the confusion.  

**Comment 5: **Please explain with concrete examples the theoretical base of the mathematical model.

**Response: **Thank you so much. We agreed with these Comments and duly explained the theoretical base of the mathematical model.

**Comment 6: ** Light up the term “green infrastructure”.

**Response:** Thank you so much for this suggestion. We followed the comments accordingly in the revised version.

# Peer review: Reviewer response for version 1

**DOI:** 10.5256/f1000research.126380.r137831

**Published:** 2022-05-23

**Authors:** Mui Yee Cheok

**Affiliations:** 1 Universiti Tun Abdul Razak Kuala Lumpur, Kuala Lumpur, Malaysia

After reviewing the research paper and other reviewers' comments I agree with the reviewers and especially many important points raised by reviewer 1, However, the following are the comments and suggestions from my side to the authors. The authors should incorporate the following suggestions and revise the manuscript.

1. The abstract needs revision and restructuring. The abstract should be revised by adding the objectives, conclusions and future scope of the proposed work.
2. Word “Externalities” is repeatedly used in the title as well as in the abstract it is not defined and the term is required to explain in the introduction
3. Market uncertainty is a wide concept that is required to explain in this paper’s context.
4. It is advised to add more literature data in the introduction section to understand the research problem, The research contributions of the present work in the existing body of knowledge should be summarized in the introduction section.
5. Paper novelty & research objectives should be added at the end of section 1 (Introduction).
6. It is advised to explain the findings of Table 1 in the text, similar all figures should be discussed in the discussion section and support some of your important findings with the past studies.

Is the work clearly and accurately presented and does it cite the current literature?

Yes

If applicable, is the statistical analysis and its interpretation appropriate?

Partly

Are all the source data underlying the results available to ensure full reproducibility?

Yes

Is the study design appropriate and is the work technically sound?

Yes

Are the conclusions drawn adequately supported by the results?

Yes

Are sufficient details of methods and analysis provided to allow replication by others?

Yes

Reviewer Expertise:

NA

I confirm that I have read this submission and believe that I have an appropriate level of expertise to confirm that it is of an acceptable scientific standard, however I have significant reservations, as outlined above.

# Response

**Published:** 2022-05-28

**Authors:** Guldana Kuandykovna Suyendikova

**Affiliations:** Department of Economics, L.N. Gumilyov Eurasian National University, Russian Federation

Dear Mui Yee Cheok

 Thank you for your valuable comments on our manuscript ‘ *Sustainable development of smart cities and smart territories based on the model of minimizing externalities’* Your recommendations have been immensely helpful, and we also appreciate your full consideration to our paper.

 As per your instructions, we have included all of your comments point by point individually, indicating exactly how we addressed each concern or problem and describing the changes we have made. The revisions have been approved by all four authors and I have again been chosen as the corresponding author. The revised manuscript was formatted according to the journal formatting criteria. We hope the revised manuscript will better suit F1000Research but are happy to consider further revisions if any, and we thank you for your interest in our research.

**REVIEWER COMMENTS, AUTHOR RESPONSES AND MANUSCRIPT CHANGES**

**Comment 1: **The abstract needs revision and restructuring. The abstract should be revised by adding the objectives, conclusions and future scope of the proposed work.

**Response: **Thank you! We have restructured the abstract in the revised version.

**Comment 2: **Word “Externalities” is repeatedly used in the title as well as in the abstract it is not defined and the term is required to explain in the introduction.

**Response: **Thank you for the identification of this confusion. The explanation was provided in the revised version.

**Comment 3**: Market uncertainty is a wide concept that is required to explain in this paper’s context.

**Response:** Thank you for highlighting this confusion. The explanation was duly provided.

**Comment 4: **It is advised to add more literature data in the introduction section to understand the research problem, The research contributions of the present work in the existing body of knowledge should be summarized in the introduction section.

**Response: **Here by investigation, we added the description in the introduction.

**Comment 5: **Paper novelty & research objectives should be added at the end of section 1 (Introduction).

**Response: **Thank you so much. We agree to this modification. The novelty was disclosed in the introduction.

**Comment 6: ** It is advised to explain the findings of Table 1 in the text, similar all figures should be discussed in the discussion section and support some of your important findings with the past studies.

**Response:** Thank you so much for this suggestion. We followed the comments accordingly in the revised version.

# Peer review: Reviewer response for version 1

**DOI:** 10.5256/f1000research.126380.r137833

**Published:** 2022-05-20

**Authors:** Jakub Kubiczek

**Affiliations:** 1 Department of Economic and Financial Analysis, University of Economics in Katowice, Katowice, Poland

Dear Authors, I am grateful for the opportunity to read and review your text.

 In my opinion, there are many approaches to your research problem, but this is the science of finding and proposing new one. I believe that the text is written correctly - although a few adjustments need to be made. I agree with reviewer 1 in many respects, but I believe that these amendments are not too complex and none of them disparages your text. You can find detailed comments below.

 Technical issues related to the structure of the article

1. Shorten the abstract to make it more specific. I would remove the introduction to the problem because the abstract should contain the essence itself. At the same time, I would add the most important results of the study.
2. “As a rule, such analysis uses the methods of surrogate markets. In turn, this direction, which is usually referred to as an indirect market method or proxy market, is divided into several pricing principles ". You write “usually” - it would be useful to support it with some other article.
3. You have defined the research gap very well and proposed a way to fill it, but there is no clearly defined purpose of the article.
4. In the results section, "the Nash equilibrium condition" appears - it would be nice to mention what it means somewhere.
5. The figures are not signed - "Source: own study."
6. Please, number the patterns continuously - they should all have a number.
7. Please, divide the conclusions into certain sections as this is too chaotic at the moment.

Substantive issues

1. The authors mention the pandemic, but do not detail how it affected the externalities. Which externalities are connected with the post-pandemic situation?
2. The authors should clarify suggesting a mathematical model as part of the management decision support.
3. I miss the justification that the presented model has potential. Therefore, I propose to note the potential of proposed model and its practical implication opportunities.
4. Do the authors mean that the nature of a sustainable development is connected with the smart territories’ development. If so, in what way? Please refer it to the proposed model.
5. Are there any recommendations for authorities and governments based on the developed model?
6. Do the researchers propose making urbanization on the basis of sustainable development?
7. Are the principles of formalization used as the basis for a mathematical model?
8. Could the developed model be qualified as the optimization of the amount of expenses that must be borne by various members of the business community?

Is the work clearly and accurately presented and does it cite the current literature?

Yes

If applicable, is the statistical analysis and its interpretation appropriate?

Partly

Are all the source data underlying the results available to ensure full reproducibility?

Partly

Is the study design appropriate and is the work technically sound?

Partly

Are the conclusions drawn adequately supported by the results?

Yes

Are sufficient details of methods and analysis provided to allow replication by others?

Partly

Reviewer Expertise:

Decision making, Corporate Social Responsibility, Sustainable Development

I confirm that I have read this submission and believe that I have an appropriate level of expertise to confirm that it is of an acceptable scientific standard, however I have significant reservations, as outlined above.

# Response

**Published:** 2022-05-28

**Authors:** Guldana Kuandykovna Suyendikova

**Affiliations:** Department of Economics, L.N. Gumilyov Eurasian National University, Russian Federation

Dear Jakub Kubiczek

 Thank you for your important comments on our manuscript ‘ *Sustainable development of smart cities and smart territories based on the model of minimizing externalities’* Your recommendations have been immensely helpful, and we also appreciate your full consideration to our paper.

 As per your instructions, i have included all of your comments point by point individually, indicating exactly how we addressed each concern or problem and describing the changes we have made. The revisions have been approved by all four authors and I have again been chosen as the corresponding author. The revised manuscript was formatted according to the journal formatting criteria. We hope the revised manuscript will better suit F1000Research but are happy to consider further revisions if any, and we thank you for your interest in our research.

**REVIEWER COMMENTS, AUTHOR RESPONSES AND MANUSCRIPT CHANGES**

**Comment 1: **Shorten the abstract to make it more specific. I would remove the introduction to the problem because the abstract should contain the essence itself. At the same time, I would add the most important results of the study.

**Response: **Thank you! We have made the relevant changes in the revised version.

**Comment 2: **The reviewer asked for the methods of the following:

 “As a rule, such analysis uses the methods of surrogate markets. In turn, this direction, which is usually referred to as an indirect market method or proxy market, is divided into several pricing principles ". You write “usually” - it would be useful to support it with some other article.

**Response: **Thank you for the identification of this confusion. The appropriate explanation was provided in the revised version.

**Comment 3**: You have defined the research gap very well and proposed a way to fill it, but there is no clearly defined purpose of the article.

**Response:** Thank You for highlighting this confusion. This confusion was removed and the purpose was written.

**Comment 4: **In the results section, "the Nash equilibrium condition" appears - it would be nice to mention what it means somewhere.

**Response: **We described the mentioned approach more precisely.  

**Comment 5: **The figures are not signed - "Source: own study.".

**Response: **Thank you so much. We agree to this modification. The figures are duly signed.

**Comment 6: ** Please, number the patterns continuously - they should all have a number.

**Response:** Thank you so much for this suggestion and modification. We followed the comments accordingly in the revised version.

**Comment 7: **Please, divide the conclusions into certain sections as this is too chaotic at the moment.

**Response: **Yes by this we mean logical explanation. Changes made accordingly in the revised version.

**Comment 8:** The authors mention the pandemic, but do not detail how it affected the externalities. Which externalities are connected with the post-pandemic situation?

**Response: **Thank you. We explained the externalities accordingly.

**Comment 9: **The authors should clarify suggesting a mathematical model as part of the management decision support.

**Response:** Thank you. We explained the mathematical model as part of the management decision support.

**Comment 10:** I miss the justification that the presented model has potential. Therefore, I propose to note the potential of proposed model and its practical implication opportunities.

**Response:** Thank you so much for identifying this confusion. We had added the potential of the model. The same has been elaborated well in the revised version.

**Comment 11: **Do the authors mean that the nature of a sustainable development is connected with the smart territories’ development. If so, in what way? Please refer it to the proposed model.

**Response: **We mean that the nature of a sustainable development is connected with the smart territories’ development.

**Comment 12: **Are there any recommendations for authorities and governments based on the developed model?

**Response: **This has been explained in the revised version.

**Comment 13:** Do the researchers propose making urbanization on the basis of sustainable development?

**Response:** Thank you so much for identifying this confusion. We had added the explanation of the model in the revised version.

**Comment 14: **Are the principles of formalization used as the basis for a mathematical model?

**Response: **We mean that the principles of formalization are used as the basis for a mathematical model implemented for the development of smart territories.

**Comment 15: **Could the developed model be qualified as the optimization of the amount of expenses that must be borne by various members of the business community?

**Response: **This has been explained in the revised version.

# Peer review: Reviewer response for version 1

**DOI:** 10.5256/f1000research.126380.r137829

**Published:** 2022-05-19

**Authors:** Bilal Khalid

**Affiliations:** 1 KMITL Business School, King Mongkut's Institute of Technology Ladkrabang, Bangkok, Thailand

**Abstract:**

- The background section of the abstract is too long, the authors should consider revising it to not more than two sentences. Because of the length of the background, the authors did not provide much information about the study in the abstract. The part above where it says “This study takes into account….” Is ideally where the authors start talking about this study. The other parts above it should be condensed into two sentences.
- The abstract should be written with the following in mind, aim/problem, objective(s), theory, methods, results, discussion, conclusions and limitations. These aspects where applicable should be touched briefly in the abstract.
- Externalities seem to be a very important concept in this study, yet it only appears once towards the end of the abstract. This is part of the problem being studied and should be reflected earlier in the abstract.

**Introduction**:

- The authors should provide a better explanation for the term “externalities” and how it affects the conceptualization of sustainable development of smart cities and smart territories.
- Cases of the impacts of externalities on the sustainable development of smart cities and territories should be provided if they are available in studies or practical examples.
- The authors should provide clarification of what they mean by “The consequences of a social nature are much wider and deeper.” It is not enough to provide citations, a context should be provided to show the relationship between the statement and the problem to research hopes to address.
- The authors should elaborate on how market uncertainty affects the sustainable development of smart cities and territories.

**Methods**:

- The first paragraphs in the Methods section should be moved to the Introduction. This section should start from the second paragraph – “The authors developed….” The authors should expand the discussion on the Methods once the first paragraph is deleted, this enables them to discuss the Methods in detail.
- The Methods require further clarification to understand the specific correlation and regression tools to be employed.

**Results**:

- The first two paragraphs of the results section can be moved to the methods section as it seems to describe actions that will lead to results, not the findings itself. The results should only contain the derivatives from what was proposed in the Methods section.
- The second line of the third paragraph contains a spacing error, kindly correct – “…carried out inside anN- dimension….”
- Authors should present the results and provide a clearer explanation, rather than using statistical jargon and equation.

**Discussion:**

- The last sentence of the analysis of the result that informs that calculations were implemented using Microsoft Excel should be taken to the Methods section. The discussion is only for the implications of your results.
- The discussion should analyze the implication of the equations and figures presented in the results. How do they affect the sustainable development of smart cities and smart territories? Please also consider citing the following articles:
  - Chaiyasoonthorn, W., Khalid, B., & Chaveesuk, S. (2019, August). Success of Smart Cities Development with Community’s Acceptance of New Technologies. Proceedings of the 9th International Conference on Information Communication and Management. ^1^
  - Khalid, B., & Naumova, E. (2021). Digital transformation of the Russian venture ecosystem under the impact of the COVID-19. In Global Challenges of Digital Transformation of Markets (pp. 313–329). Nova Science Publishers, Inc. ^2^
- There is a little linkage between the literature and the results in the discussion section. Try to connect the literature to the discussion to understand how the findings impact smart cities and smart territories.

**Conclusion**:

- The conclusion should summarize the research, make recommendations and state the limitations of the study if any. Instead, the conclusion seems to be an extension of the discussion. “Megapolises’ appears three times in the conclusion, twice in the discussion, and once in the abstract. Throughout the manuscript, it does not appear in the introduction, methods, and results. It needs to be situated in the introduction section and methods.

Is the work clearly and accurately presented and does it cite the current literature?

Partly

If applicable, is the statistical analysis and its interpretation appropriate?

Yes

Are all the source data underlying the results available to ensure full reproducibility?

No

Is the study design appropriate and is the work technically sound?

Partly

Are the conclusions drawn adequately supported by the results?

Yes

Are sufficient details of methods and analysis provided to allow replication by others?

Partly

Reviewer Expertise:

Smart Cities, Sustainable Practices, De-carbonization, Immigrant Entrepreneurship, Global Value Chains, Industry 4.0, Digital Transformations, Technology Adoption, Consumer behavior, Public Policy,

I confirm that I have read this submission and believe that I have an appropriate level of expertise to confirm that it is of an acceptable scientific standard, however I have significant reservations, as outlined above.

## References

[1] : Success of Smart Cities Development with Community’s Acceptance of New Technologies. *Proceedings of the 9th International Conference on Information Communication and Management* .2019; 10.1145/3357419.3357440 106-111 10.1145/3357419.3357440

[2] : Digital transformation of the Russian venture ecosystem under the impact of the COVID-19. In: de la Poza E, Barykin S.E. (eds): Global Challenges of Digital Transformation of Markets. *Nova Science Publishers, Inc* .2021;313-329

# Response

**Published:** 2022-05-28

**Authors:** Guldana Kuandykovna Suyendikova

**Affiliations:** Department of Economics, L.N. Gumilyov Eurasian National University, Russian Federation

Dear Dr Bilal Khalid

 Thank you for your constructive comments on our manuscript ‘ *Sustainable development of smart cities and smart territories based on the model of minimizing externalities’* The suggestions offered by you have been immensely helpful, and we also appreciate your full consideration to our paper.

 As per Your instructions, I have included all of Your comments point by point individually, indicating exactly how we addressed each concern or problem and describing the changes we have made. The revisions have been approved by all four authors and I have again been chosen as the corresponding author. The revised manuscript was formatted according to the journal formatting criteria. We hope the revised manuscript will better suit F1000Research but are happy to consider further revisions if any, and we thank you for your interest in our research.

**REVIEWER COMMENTS, AUTHOR RESPONSES AND MANUSCRIPT CHANGES**

**Comment 1: **The background section of the abstract is too long, the authors should consider revising it to not more than two sentences. Because of the length of the background, the authors did not provide much information about the study in the abstract. The part above where it says “This study takes into account….” Is ideally where the authors start talking about this study. The other parts above it should be condensed into two sentences.

**Response: **Thank you for the identification of this confusion. We have restructured the abstract. The sentences were shortened.

**Comment 2: **The abstract should be written with the following in mind, aim/problem, objective(s), theory, methods, results, discussion, conclusions and limitations. These aspects where applicable should be touched briefly in the abstract.

**Response: **Thank you! The aim, methods, results and conclusion were provided in the revised version.

**Comment 3**: Externalities seem to be a very important concept in this study, yet it only appears once towards the end of the abstract. This is part of the problem being studied and should be reflected earlier in the abstract.

**Response:** Thank you for highlighting this issue. This confusion was removed and the mentioned term was duly placed in the beginning of the abstract.

**Comment 4: **The reviewer asked about the following:

 The authors should provide a better explanation for the term “externalities” and how it affects the conceptualization of sustainable development of smart cities and smart territories.

**Response: **Here by investigation we explained this term in the introduction in the revised version in order to clarify the confusion.  

**Comment 5: **Cases of the impacts of externalities on the sustainable development of smart cities and territories should be provided if they are available in studies or practical examples.

**Response: **Thank you so much. We agree to this modification. The explanation was disclosed with more details in the introduction.

**Comment 6: ** The authors should provide clarification of what they mean by “The consequences of a social nature are much wider and deeper.” It is not enough to provide citations, a context should be provided to show the relationship between the statement and the problem to research hopes to address.

**Response:** Thank you so much for this suggestion and modification. We followed the comments accordingly in the revised version.

**Comment 7: **The authors should elaborate on how market uncertainty affects the sustainable development of smart cities and territories.

**Response: **Yes by this we mean that market uncertainty affects the sustainable development of smart cities and territories. Changes made accordingly in the revised version.

**Comment 8:** The first paragraphs in the Methods section should be moved to the Introduction. This section should start from the second paragraph – “The authors developed….” The authors should expand the discussion on the Methods once the first paragraph is deleted, this enables them to discuss the Methods in detail.

**Response: **Thank you. We made the relevant amendments.

**Comment 9: **The Methods require further clarification to understand the specific correlation and regression tools to be employed.

**Response:** We changed the manuscript accordingly. It is corrected now and all clarification was provided in the revised version.

**Comment 10** **:** The first two paragraphs of the Results section can be moved to the methods section as it seems to describe actions that will lead to results, not the findings themselves. The results should only contain the derivatives from what was proposed in the Methods section.

**Response:** Thank you so much for identifying this confusion. We had moved this sentence in the revised version.

**Comment 11** **: **The second line of the third paragraph contains a spacing error, kindly correct – “…carried out inside anN- dimension….”

**Response: **Changes made in the revised version.

**Comment 12: **Authors should present the results and provide a clearer explanation, rather than using statistical jargon and equation.

**Response: **This has been provided in the revised version.

**Comment 13** **: **The last sentence of the analysis of the result that informs that calculations were implemented using Microsoft Excel should be taken to the Methods section. The discussion is only for the implications of your results.

**Response: **The appropriate changes were made in the revised version.

**Comment 14: **The discussion should analyze the implication of the equations and figures presented in the results. How do they affect the sustainable development of smart cities and smart territories? Please also consider citing the following articles:

 Chaiyasoonthorn, W., Khalid, B., & Chaveesuk, S. (2019, August). Success of Smart Cities Development with Community’s Acceptance of New Technologies. Proceedings of the 9th International Conference on Information Communication and Management.1

 Khalid, B., & Naumova, E. (2021). Digital transformation of the Russian venture ecosystem under the impact of the COVID-19. In Global Challenges of Digital Transformation of Markets (pp. 313–329). Nova Science Publishers, Inc.

**Response: **The mentioned sources have been added in the revised manuscript.

**Comment 15: **There is a little linkage between the literature and the results in the discussion section. Try to connect the literature to the discussion to understand how the findings impact smart cities and smart territories.

**Response: **We made these changes in the revised version.

**Comment 16** **: **The conclusion should summarize the research, make recommendations and state the limitations of the study if any. Instead, the conclusion seems to be an extension of the discussion. “Megapolises’ appears three times in the conclusion, twice in the discussion, and once in the abstract. Throughout the manuscript, it does not appear in the introduction, methods, and results. It needs to be situated in the introduction section and methods.

**Response: **The conclusion was revised by your comments.
